# Ly49C-Dependent Control of MCMV Infection by NK Cells Is *Cis*-Regulated by MHC Class I Molecules

**DOI:** 10.1371/journal.ppat.1004161

**Published:** 2014-05-29

**Authors:** Catherine A. Forbes, Anthony A. Scalzo, Mariapia A. Degli-Esposti, Jerome D. Coudert

**Affiliations:** 1 Centre for Experimental Immunology, Lions Eye Institute, Nedlands, Western Australia, Australia; 2 Centre for Ophthalmology and Vision Science, M517, University of Western Australia, Crawley, Western Australia, Australia; University of California, San Francisco, United States of America

## Abstract

Natural Killer (NK) cells are crucial in early resistance to murine cytomegalovirus (MCMV) infection. In B6 mice, the activating Ly49H receptor recognizes the viral m157 glycoprotein on infected cells. We previously identified a mutant strain (MCMV^G1F^) whose variant m157 also binds the inhibitory Ly49C receptor. Here we show that simultaneous binding of m157 to the two receptors hampers Ly49H-dependent NK cell activation as Ly49C-mediated inhibition destabilizes NK cell conjugation with their targets and prevents the cytoskeleton reorganization that precedes killing. In B6 mice, as most Ly49H^+^ NK cells do not co-express Ly49C, the overall NK cell response remains able to control MCMVm157^G1F^ infection. However, in B6 Ly49C transgenic mice where all NK cells express the inhibitory receptor, MCMV infection results in altered NK cell activation associated with increased viral replication. Ly49C-mediated inhibition also regulates Ly49H-independent NK cell activation. Most interestingly, MHC class I regulates Ly49C function through *cis*-interactions that mask the receptor and restricts m157 binding. B6 Ly49C Tg, β2m ko mice, whose Ly49C receptors are unmasked due to MHC class I deficient expression, are highly susceptible to MCMVm157^G1F^ and are unable to control a low-dose infection. Our study provides novel insights into the mechanisms that regulate NK cell activation during viral infection.

## Author Summary

We previously identified a viral murine cytomegalovirus (MCMV) strain whose variant m157 immunoevasin can bind the inhibitory Ly49C NK cell receptor in addition to activating Ly49H receptor in B6 mice. Here we show that simultaneous engagement of the two receptors by m157 hampers NK cell activation. Most Ly49H^+^ NK cells lack Ly49C in B6 mice, as a result, NK cell population efficiently controls MCMV infection; however, the anti-viral response is reduced in transgenic mice where all NK cells express Ly49C. MHC I masks Ly49C through *cis*-interactions, which restricts its inhibitory function. Indeed, B6 Ly49C Tg, β2m ko mice, that are deficient for MHC I, are highly susceptible to low dose of MCMV. Our findings indicate that both the NK cell repertoire and MHC I molecules control susceptibility *vs* resistance to viral infections.

## Introduction

In humans, cytomegalovirus (CMV) is a pathogen responsible for causing significant mortality in immunocompromised patients [1] and in individuals lacking Natural Killer (NK) cells [2]. Mouse cytomegalovirus (MCMV) is a natural pathogen of mice. The similarities in structure and biology between human and mouse CMV make the latter a widely utilized model for human infection [3]. The study of MCMV has provided valuable insights into how the immune system responds to infection, and has helped to define the immune evasion mechanisms used by CMV to ensure that viral replication proceeds. NK cells play a crucial role in the early control of MCMV infection in resistant mouse strains; they limit viral replication and mortality during acute infection. The ability of NK cells to control viral infection is tightly regulated by their activating and inhibitory receptors [4]. Activating NK cell receptors include activating forms of killer cell immunoglobulin-like receptors (KIRs) in humans, and Ly49 receptors in mice. Both humans and mice express CD94/NKG2C which recognizes MHC class I molecules, and NKG2D which can be triggered by stress-induced ligands. NK cells also possess inhibitory receptors specific for MHC class I that permit discrimination of normal healthy cells from diseased ones, such as virus-infected cells, that display reduced MHC class I expression. These receptors include KIR in humans and members of the Ly49 family in mice, and LIR-1 and CD94/NKG2A in both species (reviewed in [5]). Inbred strains of mice express distinct NK cell receptor repertoires; NK cell receptors are encoded within a polygenic cluster in which each receptor gene is subject to polymorphism between the mouse strains; this variability results in resistance or susceptibility to specific viral infections.

Ly49H is the activating receptor responsible for resistance to MCMV infection in C57BL/6 (B6) mice [6]–[8]. Ly49H binds specifically to the m157 viral protein encoded by laboratory MCMV strains (Smith and K181) and triggers cytotoxicity and cytokine production [9], [10]. Arase et al showed that m157 binds to the inhibitory Ly49I receptor in 129/J mice, but not in B6 mice, while 129/J mice lack Ly49H [9]; this repertoire results in susceptibility to MCMV infection in the 129/J strain. In laboratory settings, immunological pressure through Ly49H was evidenced by the rapid selection of viral mutants producing m157 variants that escape recognition by this receptor [11]. Sequence analysis of m157 in a panel of MCMV isolates collected from a wild mouse population showed that only two isolates were identical to the laboratory MCMV strains (Smith and K181) [11], [12]. In addition, unlike the laboratory strains many of the viral isolates with m157 variants were able to replicate to high titers in resistant B6 mice [11]. We previously identified an MCMV strain (G1F) that was isolated from mice trapped in the wild; its m157 sequence shares over 93% homology with Smith and K181 strains but the protein displays a unusual binding profile to Ly49 receptors [13]. In addition to Ly49H, m157^G1F^ can bind Ly49C in B6 and BALB/c mice [13], [14].

Inhibitory Ly49 receptors are thought to play a crucial role during NK cell education. Mechanisms of NK cell education are still unclear and different models co-exist. The current consensus states that NK cells expressing inhibitory receptors specific for self MHC class I molecules are fully educated (“licensed” [15] or “not disarmed” [16]) and have a greater response potential than NK cell subsets that lack such receptors. However, recent studies showed that Ly49C^−^ NK cells are fully functional in B6 mice and indeed they dominate the Ly49H-dependent response to MCMV infection [17]. These results indicate that inhibition triggered by Ly49C binding to H-2 K^b^ overrides the responsive advantage gained by “licensing” and suggest that the inhibition mediated by Ly49C binding to H-2K^b^ regulates Ly49H-dependent NK cell activation. These data emphasize the need for a better understanding of the regulation of NK cells and how this impacts on NK cell function in the context of viral infection.

The high variability of the m157 sequence is not without similarities with human CMV, whose genome contains highly polymorphic loci that encode proteins (immunoevasins) with the potential to affect virulence through immune evasion [18]. The complex interactions of viral proteins with the host immune system are critical for determining a viral strain infectivity and pathogenicity. We undertook to study whether a viral immunoevasin able to bind multiple NK cell receptors can modulate the anti-viral immune response and to define the regulatory mechanisms. Our recent findings that m157^G1F^ binds to two NK cell receptors with opposite functions in B6 mice, provide a unique opportunity to study, in a natural infection model, how the integration of competing signals determines tolerance versus killing and ultimately the outcome of an important viral infection *in vivo*.

## Results

### The m157^G1F^ variant binds both Ly49H and Ly49C

**Figure 1:**
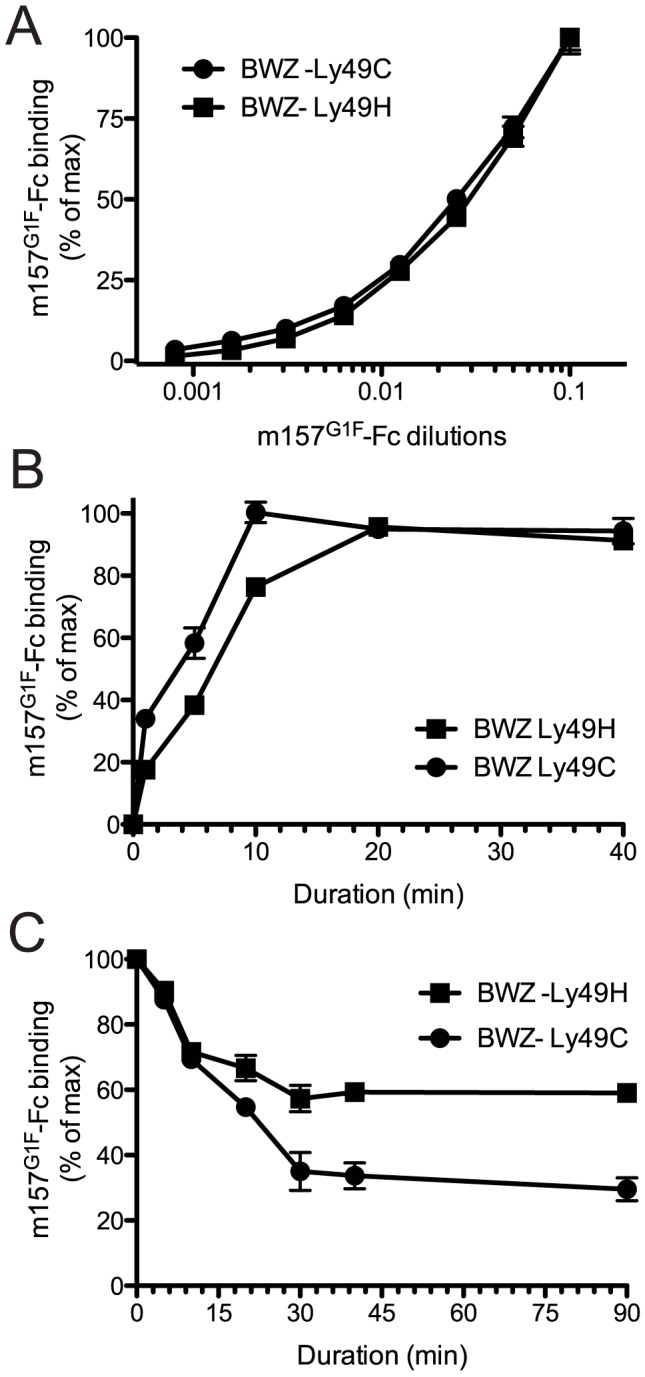
Analysis of m157^G1F^ binding to Ly49H and Ly49C expressing cells. (**A**) BWZ-Ly49H and BWZ-Ly49C cells were incubated with increasing concentrations of m157^G1F^-Fc for 40 min at 4°C and binding was measured by flow cytometry. The m157^G1F^-Fc concentration that achieved the highest mean fluorescence intensity (MFI) was used as reference (100%) and values obtained at lower concentrations were normalized according to the formula: % of binding = ((sample MFI – background MFI)/(maximal MFI – background MFI))×100). Results show MFI of triplicate values +/− SEM and are from one experiment representative of six. (**B**) BWZ-Ly49H and BWZ-Ly49C cells were incubated with saturating concentrations of m157^G1F^-Fc for periods ranging from 1 to 40 min at 4°C and the resulting binding was analyzed by flow cytometry. The MFI obtained at 40 min was used as reference (100%) and values obtained after shorter incubation times were normalized according to the formula: % of binding = ((timed MFI – background MFI)/(MFI at 40 min – background MFI))×100). Results show mean of triplicate values +/− SEM and are from one experiment representative of six. (**C**) Ly49H and Ly49C-expressing BWZ cells were labeled with saturating concentrations of m157^G1F^-Fc at 4°C; the cells were then washed and incubated at 37°C in the presence of excess concentrations of anti-m157 blocking antibody (6H121) to prevent re-binding of detached m157-Fc. A measure of the MFI prior to the addition of 6H121 provided maximal binding values (100%). Binding decay was measured at times from 5 to 90 min of incubation at 37°C. Binding decay values were calculated using the formula: % of binding = ((timed MFI – background MFI)/(MFI at 0 min – background MFI))×100). Results show mean of triplicate values +/− SEM are from one experiment representative of four.

We previously demonstrated the ability of m157 from the MCMV^G1F^ strain (m157^G1F^) to bind both Ly49H and Ly49C in B6 mice [13]. Here, we analyzed the binding properties of m157^G1F^ and aimed to determine whether it can bind the two receptors simultaneously. We transfected BWZ.36 cells to express either Ly49H^B6^ (BWZ-Ly49H) or Ly49C^B6^ (BWZ-Ly49C) and clones expressing the receptors at similar levels were selected to test m157^G1F^-Fc binding, by flow cytometry. Firstly, we measured the binding obtained at increasing concentrations of the m157^G1F^-Fc. Titration curves were similar for Ly49H and Ly49C expressing cells (Fig. 1A). No binding was detected to the parental BWZ.36 cells (data not shown). These results suggest that m157^G1F^ binds to Ly49H and Ly49C with similar affinities. Next, we compared the kinetics of binding of m157^G1F^ to these two receptors. Ly49H or Ly49C-expressing cells were incubated with saturating concentrations of m157^G1F^-Fc, as determined above, for 30 sec to 40 min. Fluorescence obtained after 40 min provided the maximal intensity value (100%), while the background to be subtracted was measured in the absence of fusion protein; the percentages of binding achieved over increasing incubation periods were calculated accordingly. Binding of m157^G1F^ to Ly49C was found to occur slightly more quickly than to Ly49H, with 50% binding reached after 5 and 7 min, respectively (Fig. 1B). Thirdly, we measured the stability of the interactions. For this purpose, we used the optimal binding conditions previously determined, and analyzed dissociation rates. Excess amounts of anti-m157 antibody (6H121) prevented re-association of the fusion proteins to the receptors after they detached. We found that 50% of m157^G1F^ that was initially bound to Ly49C had dissociated after approximately 20 min while over 50% was still attached to Ly49H after 90 min (Fig. 1C). These results suggest that m157^G1F^ dissociates more quickly from Ly49C than Ly49H. A comparative binding analysis of m157^K181^ and m157^G1F^ to Ly49H was also conducted as a control; our results showed similar binding characteristics of the two m157 variants to Ly49H (Fig. S1). This data confirmed our previous findings [13].

Overall, these results suggest that m157^G1F^ binds to Ly49H and Ly49C with similar affinities but interactions with Ly49H are more sustained.

### Binding of m157^G1F^ to Ly49C is limited by *cis*-interactions with MHC class I molecules

**Figure 2:**
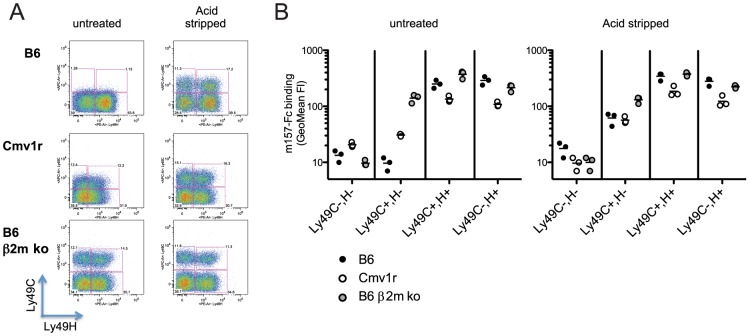
Binding of m157^G1F^ to NK cells isolated from various mouse strains. Splenic NK cells were enriched from naïve B6, Cmv1r or B6 β2m ko mice. NK cells were either untreated or acid stripped. (**A**) NK cells were incubated with fluorochrome-conjugated anti-Ly49C and Ly49H antibodies and analyzed by flow cytometry to discriminate the corresponding cell subsets. (**B**) NK cells were incubated with m157^G1F^-Fc and then with fluorochrome-conjugated antibodies to discriminate Ly49H and Ly49C NK cells subsets. The four following subsets were analyzed for m157^G1F^ binding: 1- Ly49C^−^,H^−^, 2- Ly49C^+^,H^−^, 3- Ly49C^+^,H^+^, 4- Ly49C^−^,H^+^. Horizontal bars represent the mean values of m157^G1F^ binding measured from three individual mice (each represented as a circle). Data are from one experiment representative of three performed.

We previously showed that m157^G1F^ binds to NK cells that are stained with the 5E6 mAb (*i.e.* NK cells expressing Ly49C and/or I) in B6 mice [13]. Here, we further analyzed m157^G1F^ binding to NK cell subsets expressing Ly49H and/or Ly49C. Splenic NK cells were collected from the following mouse strains: B6 (H-2^b^), Cmv1^r^ (H-2^d^ background expressing B6 alleles in the NK cell receptor gene locus) [19] and B6 β2m ko (defective for MHC class I expression). Purified NK cells were stained with Ly49C-specific 4LO3311 and Ly49H-specific 3D10 antibodies in order to discriminate the respective NK cell subsets. We used the 4LO3311 antibody that is specific for Ly49C rather than 5E6, which also recognizes Ly49I [20]. 4LO3311 failed to stain B6 NK cells while it stained weakly Cmv1^r^ NK cells; in contrast, NK cells from B6 β2m ko mice showed strong staining with 4LO3311 (Fig. 2A, left panels). In B6 mice, self-ligands for Ly49C are MHC class I molecules H-2 K^b^; Ly49C can engage H-2 K^b^ on other cells (*trans*-interaction) as well as H-2 K^b^ expressed on the same NK cell membrane plan (*cis*-interaction) [21]. *Cis*-binding of the Ly49A inhibitory receptor with MHC class I has been shown to restrict the interactions with molecules presented in *trans* [22]. Likewise, the staining patterns detected on NK cells isolated from the three mouse strains analyzed were consistent with masking of Ly49C due to *cis*-interactions with H-2 K^b^. In order to determine whether the differential staining illustrated in Fig. 2A were indeed due to *cis*-masking, we treated NK cells with an acid buffer; this treatment releases the β2m from MHC class I heavy chain, and disrupts *cis*-interactions [21], [22]. 4LO3311 bound to Ly49C on acid stripped B6 NK cells, while a more intense staining was achieved on Cmv1^r^ NK cells; binding to B6 β2m ko NK cells remained unchanged (Fig. 2A, right panels). These results indicated that Ly49C is masked due to *cis*-interactions with MHC class I molecules in B6 mice. The existence of a partial masking in Cmv1^r^ suggests that Ly49C also binds to H-2^d^ MHC class I in *cis* but with a lower affinity than to H-2^b^ molecules.

Next, we analyzed the binding of m157^G1F^ to NK cell subsets expressing Ly49H and/or Ly49C (Fig. 2B). Using B6 β2m ko cells where Ly49C is free of *cis*-masking, m157^G1F^ binding was detected to both Ly49H and Ly49C-expressing NK cell subsets. In B6, m157^G1F^ binding was achieved only in the NK cell subsets expressing Ly49H, while in Cmv1^r^, m157^G1F^ displayed weak binding to the Ly49C^+^, Ly49H^−^ (Ly49C^+^,H^−^) NK cell subset. Acid stripping improved m157^G1F^ binding to the Ly49C^+^,H^−^ subset from B6 and Cmv1^r^ and also resulted in enhanced binding to the subset co-expressing both receptors. The absence of m157^G1F^ binding to untreated Ly49H^−^ NK cells, despite a fraction of these cells expressing Ly49C, indicated that m157^G1F^ cannot bind Ly49C when interactions in *cis* made it unavailable. As a control, we also incubated untreated and acid treated NK cells with the secondary antibody and with streptavidin in the absence of the first m157-Fc incubation step. We did not detect any fluorescence above the background, indicating the specificity of our multi-step staining in both untreated and acid treated NK cells (Fig. S2).

Consistent with the data we obtained using cell lines expressing NK cell receptors (Fig. 1), these results indicate that m157^G1F^ can bind to NK cells expressing Ly49C and/or Ly49H. However, the ability to engage Ly49C is limited by MHC class I molecules (H-2^b^>H-2^d^) due to *cis* interactions.

### Ly49C can limit Ly49H-dependent activation by m157

**Figure 3:**
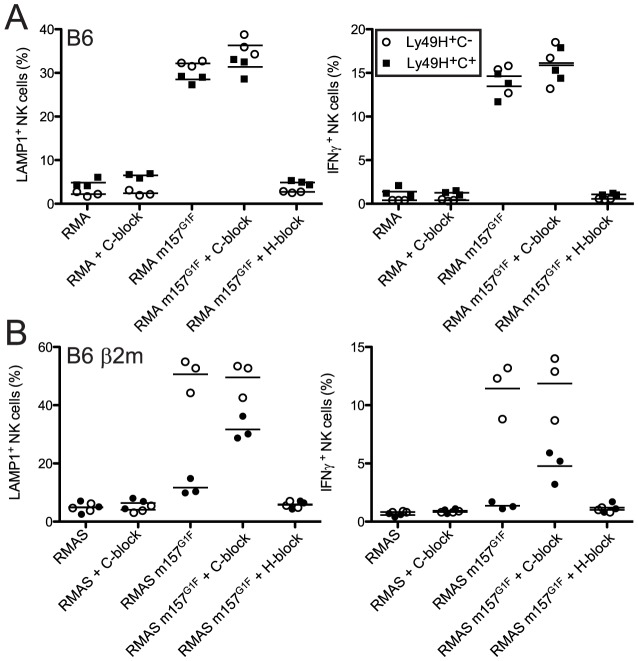
m157^G1F^–Ly49C binding regulates Ly49H-dependent NK cell functions *ex vivo*. Splenic NK cells were enriched from naïve B6 wt (**A**) or B6 β2m ko (**B**) mice and exposed to parental or m157^G1F^ expressing RMA (**A**) or RMAS cells (**B**). Excess concentrations of anti-Ly49C (4LO3311) or Ly49H (3D10) were added where indicated to block the corresponding receptors. At the end of the coculture, cells were stained with fluorochrome-conjugated antibodies to discriminate NK cell subsets (Ly49H^+^,C^−^ (open circles) and the Ly49H^+^,C^+^ (filled squares)) and detect degranulation (LAMP-1, left panels) and IFN-γ production (right panels). Horizontal bars represent the mean values from three individual mice (each represented as a symbol). These data are from one experiment representative of three performed.

We previously demonstrated that m157^G1F^ induces intracellular signals upon binding to both Ly49H and Ly49C [14]. Here, we analyzed whether m157^G1F^ differentially activates NK cells that express Ly49H only or coexpress Ly49H and Ly49C. B6 NK cells were exposed to target cells expressing m157^G1F^ (RMA m157^G1F^) or to parental RMA cells and analyzed for degranulation and production of IFN-γ (Fig. 3A). Upon exposure to RMA m157^G1F^, Ly49H^+^,C^−^ NK cells degranulated (∼40% LAMP1^+^) and produced IFN-γ (∼15% IFN-γ^+^). NK cell activation was Ly49H-dependent as it was abolished in the presence of blocking 3D10 antibodies. However, co-expression of Ly49C did not alter the response. We hypothesized that *cis*-masking of Ly49C by H-2 K^b^ was responsible for the absence of inhibition. We therefore examined NK cells isolated from B6 β2m ko mice, where Ly49C receptors are not masked in *cis* (Fig. 2A). We used RMAS cells (MHC class I deficient) expressing m157^G1F^ (RMAS m157^G1F^) as targets instead of RMA m157^G1F^ cells in order to prevent competition between m157 and K^b^ for binding to Ly49C, thus, ensuring that inhibition through Ly49C would only be due to m157 binding. RMAS m157^G1F^ cells strongly activated Ly49H^+^,C^−^ NK cells but not the Ly49H^+^,C^+^ subset. As above, activation was Ly49H-dependent. Blocking of Ly49C enabled Ly49H^+^,C^+^ NK cell activation, albeit not as strongly as the Ly49H^+^,C^−^ subset (Fig. 3B).

Therefore, NK cell exposure to m157^G1F^-expressing cells triggers optimal Ly49H-dependent NK cell functions when Ly49C is not co-engaged. These results demonstrate that Ly49C can restrict Ly49H-dependent activation upon engagement of m157, but this inhibition is itself regulated *in cis* by MHC class I molecules.

### Ly49C dampens NK cell activation by inhibiting cytoskeleton polymerization

**Figure 4:**
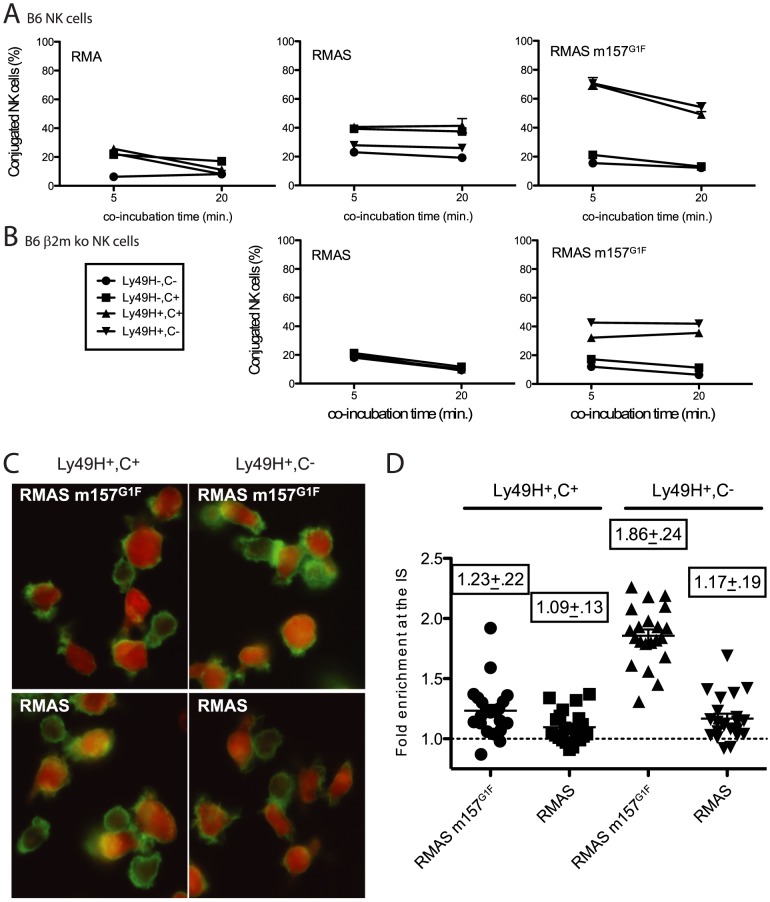
m157^G1F^ hampers NK cell-target conjugate formation and cytoskeleton polymerisation. IL-2 expanded NK cells isolated from B6 wt (**A**) and B6 β2m ko mice (**B**) were sorted into four subsets according to Ly49H and Ly49C expression (1- Ly49H^−^,C^−^; 2- Ly49H^−^,C^+^; 3- Ly49H^+^,C^+^; and 4- Ly49H^+^,C^−^. They were further expanded in IL-2 containing medium to restore expression of antibody-free receptors before being tested for conjugate formation with the following target cells: RMA, RMAS or RMAS m157^G1F^. NK cells were labelled with CMTMR and targets with CFSE, then mixed and incubated for 5 or 20 min at 37°C. Percentages of conjugated NK cells were determined by flow cytometry. (**C**) Sorted B6 β2m ko NK cells (Ly49H^+^,C^+^: left panels; Ly49H^+^,C^−^: right panels) were exposed to CMTMR-labelled RMAS (bottom panels) or RMASm157^G1F^ (top panels) for 15 min at 37°C. Polymerised cytoskeleton actin was detected using phalloidin-FITC and fluorescence microscopy analysis. (**D**) Enrichment of cytoskeleton F-actin at the cell contact area was quantified in the samples described in **C** using *ImageJ* software. Horizontal bars represent the mean of individual enrichment values (each symbol illustrates an individual conjugate). Mean values +/− SD for each condition tested are indicated above the symbols. These data are from one experiment representative of three performed.

Next we evaluated whether m157 binding to Ly49C affects the stability of cell conjugates formed between NK cells and their targets thereby preventing subsequent killing. B6 NK cells were expanded in IL2-containing medium and four subsets were sorted based on Ly49C and Ly49H expression (1. Ly49H^−^,C^−^, 2. Ly49H^−^,C^+^, 3. Ly49H^+^,C^+^ and 4. Ly49H^+^,C^−^). After further expansion, to eliminate residual fluorescence resulting from the sorting step, NK cells and target cells were labeled using intracellular dyes, CFSE and CMTMR respectively. NK cells were then exposed to various target cells and the formation of stable cell conjugates was assessed. Because B6 NK cells interactions with RMA cells do not result in killing, RMA cells were used as negative controls. RMA m157^G1F^ were excluded as targets because they express K^b^ that can bind Ly49C and it would have been impossible to determine whether the effects of Ly49C on conjugate stability were due to engagement of m157 or K^b^. Instead, we used MHC class I-deficient RMAS m157^G1F^ cells. RMAS cells were also tested; they are killed by B6 NK cells due to “missing self recognition”, as described by [23], which is Ly49H-independent. Each of the four NK cell subsets indicated in Fig. 4A was exposed to target cells for 5 and 20 min and then the percentage of conjugated NK cells was analyzed by flow cytometry. In all four subsets, the fraction of NK cells conjugated with RMA cells remained below 20% (Fig. 4A, left panel). Using RMAS as targets, conjugation levels with NK cells expressing Ly49C increased to 40%, while they remained around 20% with the Ly49C^−^ NK cells (Fig. 4A, middle panel). In B6 mice, Ly49C is involved in NK cell education [15], its expression resulting in a higher reactivity. This is consistent with the increased conjugate rates obtained when Ly49C^+^ NK cell subsets were exposed to RMAS cells. Interestingly, increased formation of conjugates by Ly49H^−^,C^+^ NK cells with RMAS (up to 40%, Fig. 4 A middle panel) were abolished when this subset was exposed to RMAS m157^G1F^ (Fig. 4A right panel). This suggests that binding of m157^G1F^ to Ly49C compensated for the absence of K^b^ and prevented “missing-self recognition”. Incubation of Ly49H^+^ NK cells with RMAS m157^G1F^ resulted in high conjugation rates regardless of whether Ly49C was co-expressed (Fig. 4A, right panel). We hypothesized that the similar frequencies of cell conjugates detected in Ly49H^+^,C^+^ and Ly49H^+^,C^−^ B6 NK cells with RMAS m157^G1F^ (which suggested an absence of inhibitory effect mediated by Ly49C) was due to *cis*-masking by MHC class I molecules. Therefore, we isolated NK cells from B6 β2m ko mice to address this hypothesis. Although β2m ko mice are deficient for MHC class I expression, their NK cells undergo an education process that enables them to respond to MCMV infection as efficiently as wild type mouse cells (our unpublished results and [24]), while they are tolerant toward cells displaying altered MHC I expression. As described above, we sorted four NK cell subsets and exposed them to RMAS and RMAS m157^G1F^ cells. The percentages of NK cells conjugated to RMAS cells remained around 20% for the four subsets. This result was consistent with NK cell tolerance toward MHC class I-devoid RMAS cells (Fig. 4B, left panel) in β2m ko mice. Incubation of RMAS m157^G1F^ with the subsets expressing Ly49H resulted in increased conjugates, although to a lesser extent than seen with B6 NK cells (Fig. 4A and B). In addition, the Ly49H^+^,C^+^ NK cell subset demonstrated a reproducibly lower conjugation rate with RMAS m157^G1F^ than the subset expressing Ly49H only (Fig. 4B, right panel).

These results suggest that “missing-self recognition” and Ly49H-dependent signals synergize, resulting in more conjugated NK cells (70% when NK cells were from B6 wt mice, Fig. 4A), while the interactions exclusively due to missing self-recognition (Fig. 4A middle panel) or to Ly49H engagement (Fig. 4B right panel) resulted in more modest conjugation rates (40%). Thus, m157^G1F^ binding to Ly49C disrupts the cell conjugates formed in the context of “missing self recognition”, but a simultaneous engagement of Ly49H can override this inhibition.

Interactions between NK cells and target cells that trigger killing events require the formation of stable cell conjugates [25]. During this process, cytoskeleton polymerization orients the cytotoxic granules toward the immunological synapse. Polymerization of the cytoskeleton actin into F-actin can be detected by microscopy using fluorescent phalloidin. B6 β2m ko NK cells were sorted according to their Ly49H and Ly49C expression as above, then were incubated with CMTMR-labeled RMAS or RMAS m157^G1F^ cells and tested for actin polymerization. NK cell subsets expressing Ly49H but not Ly49C displayed a strong polarization of their cytoskeleton toward RMAS m157^G1F^ (fig. 4C, upper right panel) whereas NK cells co-expressing Ly49H and Ly49C did not (fig. 4C, upper left panel). This process was m157-dependent as incubation with parental RMAS cells did not trigger actin polymerization (fig. 4C, lower panels). Quantification of F-actin within the immunological synapse further supported this result, confirming cytoskeleton accumulation toward m157^G1F^ expressing cells within the Ly49H^+^,C^−^ NK cell subset (fig. 4D). Thus, m157 binding to Ly49C reduces the level of cytoskeleton actin polymerization and accumulation toward the target cell.

### Replication of MCMV m157^G1F^ in Ly49H^+^ mice

**Figure 5:**
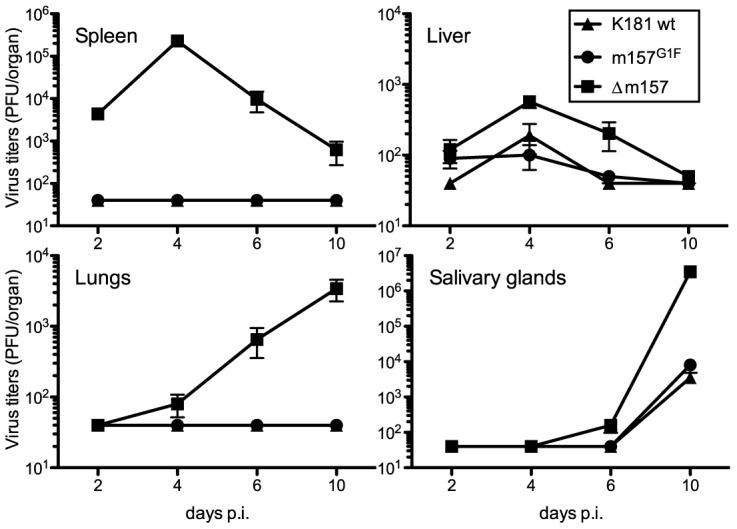
Replication kinetics of MCMVm157^G1F^ in Cmv1r mice. Cmv1r mice were infected with 1×10^4^ PFU of MCMV K181 wt (triangles), MCMVm157^G1F^ (circles) or MCMVΔm157 (squares) viruses and the organs indicated were harvested at days 2, 4, 6 and 10 p.i. Viral titers were determined from organ homogenates by standard plaque assay. Bars represent average values in each organ collected from 3 mice per group +/− SEM. Data are from one experiment representative of two performed.

We previously showed that the MCMV K181 laboratory strain in which m157 was substituted by m157^G1F^ (MCMV m157^G1F^), replicates in B6 mice at a similar rate to the MCMV K181 wt virus [13]. In B6 mice, Ly49C is mostly unavailable for binding m157 due to *cis*-interaction with H-2 K^b^ molecules, as illustrated in Fig. 2. We analyzed MCMV m157^G1F^ replication in a H-2^d^ background using Cmv1^r^ mice (Ly49H^+^) where *cis*-interactions are weak (Fig. 2). Mice were infected with either MCMV K181 wt, MCMV m157^G1F^ or with a recombinant virus with m157 deleted (MCMV Δm157) and measured viral titers in the spleen, liver, lungs and salivary glands at various times post infection. Viral loads we measured in Cmv1^r^ mice were similar to those observed previously in B6 mice (Fig. 5 and [13]). Only MCMV Δm157, which escapes NK cell surveillance, replicated at high titers while MCMV K181 wt and MCMV m157^G1F^ were similarly controlled. A possible explanation is that, although weak in Cmv1^r^, *cis*-masking of Ly49C restrained sufficiently its ability to engage m157^G1F^, and so impeded its inhibitory effect over Ly49H-dependent activation. In addition, as most Ly49H^+^ NK cells do not co-express Ly49C in Cmv1^r^ mice (as in B6) and therefore cannot be inhibited by m157, we proposed that this subset ensured virus elimination and compensated for a likely impaired response of the Ly49H^+^,C^+^ subset. To test this hypothesis, we generated a Ly49C transgenic mouse strain (B6 Ly49C Tg) in which all NK cells express this inhibitory receptor.

### Replication of MCMV m157^G1F^ in B6 Ly49C Tg mice

**Figure 6:**
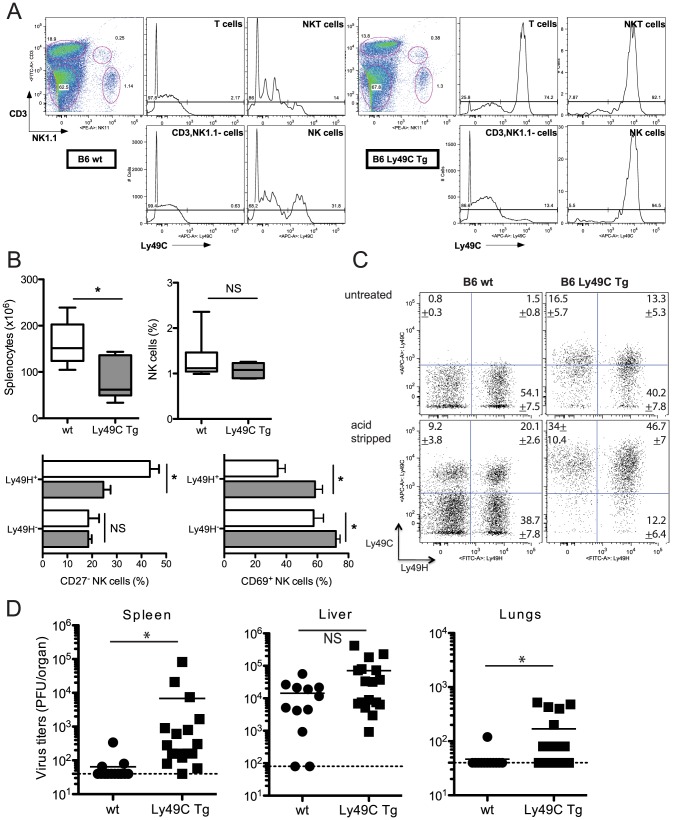
Infection of B6 Ly49C Tg mice by MCMVm157^G1F^. (**A**) Blood borne lymphocytes from Ly49C Tg B6 mice (right panels) or negative littermates (left panels) were exposed to acidic treatment and stained with fluorochrome conjugated antibodies for flow cytometric analysis. The histograms illustrate Ly49C expression in NK cells, NKT cells, T cells and CD3^−^, NK11^−^ leukocytes. (**B,C,D**) Ly49C Tg and wt B6 mice were infected with 5×10^4^ PFU of MCMVm157^G1F^. (**B.C**) Four days p.i. splenic NK cells were analysed by flow cytometry. Box and Whiskers graphs represent the range of individual mouse values spanning from 5 to 95 percentiles; they comprise 11 mice per group combining three independent experiments (**B**). (**C**) Dot plot analysis of untreated or acid stripped splenic NK cells. Values indicate percentages +/− SEM for each of the four cell subsets calculated from 11 mice per group that were analysed in three independent experiments. (**D**) Alternatively, spleen, liver and lungs were collected at d4 p.i. to measure virus titers by standard plaque assay. Horizontal bars represent the mean of individual values; each mouse is illustrated as a symbol (n = 12 B6 wt and n = 16 B6 Ly49C Tg). Differences between the groups were compared using the Mann-Whitney U test: *: p<0.05, NS: not significant).

We generated the B6 Ly49C Tg mouse strain as indicated in the Materials and Methods section. Analysis of blood-borne NK cells indicated than over 95% expressed Ly49C (Fig. 6A). Ly49C was also found in over 50% of T cells and in most NKT cells; it was also expressed in a small fraction of B cells (<20%, data not shown), but in none of the other leukocyte populations (data not shown). The transgenic mice and negative littermates had splenic and bone marrow compartments of similar size (Fig. S3). Wild-type and transgenic spleens contained comparable frequencies of B cells, NKT cells and DCs; the T cell fraction was slightly reduced in the transgenic mice, while monocytes/macrophages, neutrophils and eosinophils were slightly increased (Fig. S3). Most importantly, the size of the Ly49H^+^ NK cell subset was unchanged (Fig. S4). A fraction of Ly49C receptors was found to be masked by *cis* interactions (Fig. S4).

Ly49C Tg and negative littermates were infected with MCMV m157^G1F^ and viral titers and NK cell activation tested after 4 days. The spleens in transgenic mice were not as enlarged as in wt mice upon infection, consistent with previous studies in Ly49H-devoid mouse strains [26], [27], although the percentage of splenic NK cells was similar (Fig. 6B). The frequency of Ly49H^+^ NK cells in wt and transgenic mice were also identical (Fig. 6C). CD27 dissects peripheral NK cells into two major subsets: naïve NK cells express CD27, while this marker is downregulated in activated and more mature NK cells [28]. We found that the frequency of the CD27^−^ cells within Ly49H^+^ NK cells was higher in B6 wt than in Tg mice, while it was similar in Ly49H^−^ NK cells (Fig. 6B). This suggests that Ly49H dependent activation was reduced in Ly49C Tg mice. We also analyzed the expression of the activation marker CD69 in Ly49H positive or negative NK cells. We found that CD69 was more increased in Tg than in wt mice, most particularly in Ly49H^−^ NK cells (Fig. 6B). This sustained activation did not require Ly49H and was most likely due to higher cytokine levels associated with uncontrolled viral proliferation. Viral loads were measured in the spleen, liver and lungs after 4 days. A high dose of virus (5×10^4^ PFU) was used so that viral titers found in wt mouse tissues would be just above the detection limit. Ly49C Tg mice had increased viral titers in the spleen and lungs; there was a trend for increased hepatic loads although the difference was not statistically significant (Fig. 6D). Thus expression of Ly49C on all NK cells results in an inhibitory effect of the immunoevasin m157 leading to higher viral replication *in vivo*.

Ly49C *cis*-masking was not as complete in B6 Ly49C Tg as in wt mice (Fig. 6C); we predicted that if more Ly49C receptors were rendered available in the absence of *cis*-interactions, the inhibitory effect on NK cells would be more intense and would lead to even more severe viral replication.

### Replication of MCMV m157^G1F^ in B6 Ly49C Tg, β2m ko mice

**Figure 7:**
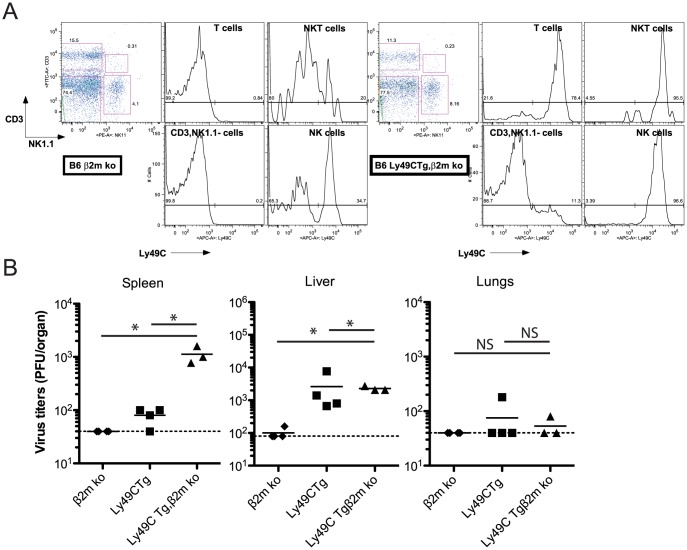
Infection of B6 Ly49C Tg β2m ko mice by MCMVm157^G1F^. (**A**) Blood borne lymphocytes from Ly49C Tg, β2m ko B6 mice (right panels) or B6 β2m ko mice (left panels) were stained with fluorochrome conjugated antibodies for flow cytometric analysis. Histograms illustrate Ly49C expression in NK cells, NKT cells, T cells and CD3^−^, NK11^−^ leukocytes. (**B**) B6 Ly49C Tg, β2m ko and B6 β2m ko mice were infected with 5×10^3^ PFU of MCMVm157^G1F^. Four days p.i. the spleen, liver and lungs were collected to measure virus titers by standard plaque assay. Horizontal bars represent the mean of individual values; each mouse is illustrated as a symbol. Horizontal bars represent the mean of individual values, each mouse is illustrated as a symbol; groups are constituted of 6–8 mice. Differences between the groups were compared using Mann-Whitney U test: *: p<0.05, NS: not significant).

We crossed B6 Ly49C Tg mice with B6 β2m ko mice and obtained B6 Ly49C Tg, β2m ko double mutant mice. These were used to test *in vivo* the anti-viral NK cell response in the absence of Ly49C *cis*-masking. As in B6 Ly49C Tg mice, expression of the Ly49C transgene in the double mutant mice was limited to NK cells, NKT cells, most T cells (Fig. 7A) and a small fraction of B cells (data not shown). Ly49C was not detected elsewhere, and had little impact on the leukocyte population sizes (Fig. S5). B6 Ly49C Tg, β2m ko mice were infected along with B6 β2m ko and B6 Ly49C Tg using 5×10^4^ PFU MCMVm157^G1F^, as previously. B6 Ly49C Tg, β2m ko mice were highly susceptible to viral infection with 3 out of 4 of these mice succumbing to infection by day 3. Infection with a lower inoculum (5×10^3^ PFU) was performed and the viral titers were measured in the spleen, liver and lungs after 4 days (Fig. 7B). Splenic viral loads were higher in B6 Ly49C Tg, β2m ko mice than in B6 Ly49C Tg mice. Viral replication was completely controlled in B6 β2m ko mice, indicating that the absence of cytotoxic CD8 T cells due to the lack of MHC class I was not responsible for the impaired immune response at this early stage of acute infection.

We concluded that inhibitory Ly49C receptor is most efficient at negatively regulating NK cell functions when it are not engaged through *cis*-interactions and that targeting of this receptor by the m157 immunoevasin severely impairs the Ly49H-dependent response.

### Inhibition of Ly49H-independent NK cell activation via m157^G1F^-Ly49C binding

Our results illustrated in Fig. 4A,B indicated that m157^G1F^ binding to Ly49C could modulate “missing self recognition”. We investigated this possibility further in TC1 congenic mice; this strain has a B6 background, but has BALB/c alleles in the Ly49 cluster of the NKC and thus lacks Ly49H, as described in [19]. Missing-self recognition dependent killing in the B6 background is mainly mediated by Ly49C^+^ NK cells [16]. We hypothesized that RMAS m157^G1F^ target cell killing by TC1 NK cells would be impaired due to Ly49C-mediated inhibition, even though the targets were devoid of MHC class I molecules.

**Figure 8:**
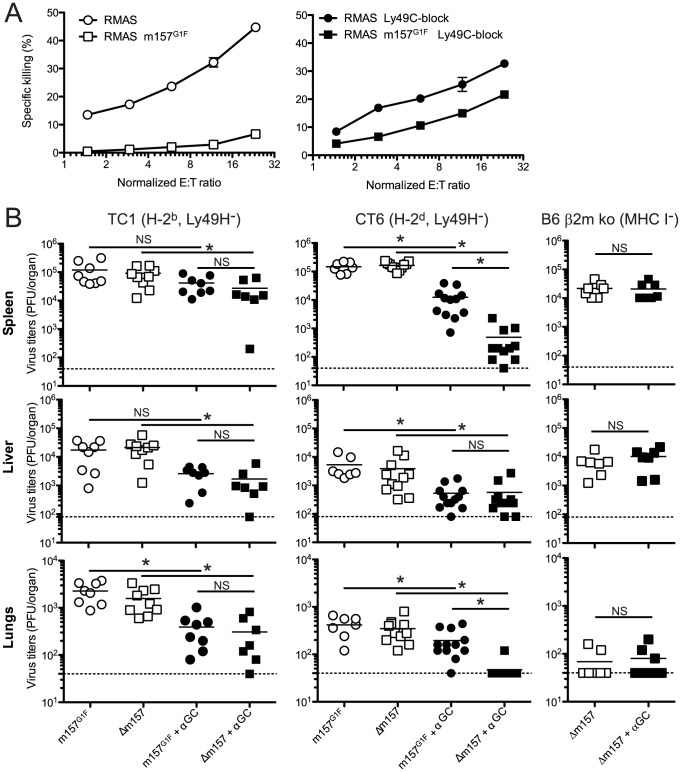
m157^G1F^ regulates Ly49H-independent NK cell activation. (**A**) Splenic NK cells were isolated from TC1 mice (H-2^b^, Ly49H^−^) and expanded in IL-2 containing medium. NK cells were left unblocked (left panel) or were incubated with anti-Ly49C (4LO3311) blocking antibody (right panel) and co-cultured for 4 h with ^51^Cr-loaded RMAS or RMAS m157^G1F^ target cells at the indicated effector∶target ratios (E∶T). Each sample was tested in triplicate. The data illustrated represent means +/− SD and are from one experiment representative of four performed. (**B**) TC1 (H-2^b^, Ly49H^−^; left panels), CT6 (H-2^d^, Ly49H^−^; middle panels) or B6 β2m ko mice (no MHC I, Ly49H^+^; right panels) were infected with 1×10^4^ PFU of MCMVm157^G1F^ or MCMVΔm157; the viruses were administered alone or in combination with 2 µg alpha-galactosylCeramide (αGC). Four days p.i., spleen, liver and lungs were collected and viral titers within these organs were measured by standard plaque assay. Bars represent mean values from 7–12 mice/group pooled from 3 independent experiments, the dotted lines represent the limit of detection. Differences between the groups were compared using the Mann-Whitney U test: *: p<0.05, NS: not significant).

TC1 NK cells were expanded in IL-2 containing medium and tested against RMAS and RMAS m157^G1F^ in an *in vitro* killing assay. As expected, NK cells killed RMAS targets. By contrast, they spared RMAS m157^G1F^ cells (Fig. 8A, left panel). Addition of excess concentrations of Ly49C-blocking antibody enabled NK cells to kill RMAS m157^G1F^ targets (Fig. 8A, right panel). These results suggest that engagement of Ly49C by m157^G1F^ induces inhibitory signals that compensate for the absence of MHC class I, and thereby prevents NK cell cytotoxicity. We then tested *in vivo* whether m157^G1F^ could affect the control of MCMV infection mediated by NK cells in a Ly49H-independent manner. Some MCMV genes down-regulate MHC class I expression in infected cells [29]. Previous studies showed that levels of expression of MHC class I can affect NK cell responses during MCMV infection [30], [31]. We hypothesized that NK cell killing of infected cells with reduced MHC class I expression would be prevented by m157^G1F^ binding to Ly49C. We infected Ly49H-devoid mice with MCMV m157^G1F^ or with MCMV Δm157 and compared the replication of the two viruses; infected mice displayed similarly high titers in the spleen, liver and lungs after 4 days (Fig. 8B). We hypothesized that strong inflammatory conditions would promote a better anti-viral NK cell response. Administration of alpha-galactosyl ceramide (αGC) at the time of infection improves NK cell mediated control of MCMV in Ly49H-deficient mice; this effect is NKT cell independent [32]. Next, we administered αGC in combination with MCMVm157^G1F^ or Δm157 and measured viral titers 4 days later. Treatment using αGC resulted in reduced replication of Δm157 virus in all organs (Fig. 8B). TC1 mice treated with αGC had similar viral titers following infection irrespective of which of the two viruses was used (Fig. 8B). We predicted that the absence of an effect associated with m157^G1F^ expression was due to *cis*-masking of Ly49C. Therefore, we repeated the experiment in CT6 mice (H-2^d^, Ly49H^−^) [19], where *cis*-interactions are weak, as shown in Fig. 2. In this H-2^d^ background, although Ly49C is not responsible for self recognition, m157^G1F^ can bind the inhibitory receptor [13] and induce inhibitory signals. Infection with the Δm157 virus combined with αGC treatment was almost completely controlled, while the protection conferred by αGC was only partial after infection with the m157^G1F^ virus. We also tested B6 β2m ko mice whose NK cell are tolerant to cells with reduced MHC class I expression and where Ly49C availability is not hampered by *cis*-masking. Only the Δm157 virus was tested in this mouse strain as the m157^G1F^ virus would have triggered a dominant Ly49H-dependent response. Contrary to those measured in TC1 mice, identical Δm157 virus titers were measured with and without αGC treatment (Fig. 8B). These results are consistent with a Ly49C-mediated inhibition of a Ly49H-independent NK cell response; it is achieved upon binding of the m157 immunoevasin and is regulated through *cis*-interactions.

Altogether, our results demonstrate that engagement of the inhibitory Ly49C receptor by the m157 immunoevasin inhibits NK cell activation leading to failure to control MCMV infection, and that Ly49C inhibitory function is modulated by MHC I molecules through *cis*-masking.

## Discussion

We previously showed, and confirmed in this study, that m157 from the K181 and the G1F MCMV strains bind Ly49H with similar affinities [13]. Here, we analyzed the kinetics of binding of m157^G1F^ to Ly49H or Ly49C. We found that the kinetics of association of m157^G1F^ to Ly49C was slightly quicker than to Ly49H. In addition, the binding profiles suggested similar affinities for the two receptors, consistent with the results obtained by surface plasmon resonance analysis [14]. Kinetics of dissociation of m157 indicated that interactions with Ly49H were more sustained. This could suggest that after some degree of initial inhibition, Ly49H activation would proceed unchallenged when NK cells coexpress the two receptors. However, since Ly49C is not internalized upon engagement, unlike Ly49H, it is likely that the Ly49C receptors that have dissociated from m157 will establish new interactions and induce inhibitory signals as long as the NK cell-target pairs remain conjugated. Nevertheless, these different dissociation rates could lead to competition in a mixed population where some NK cells express exclusively Ly49H or Ly49C that would ultimately favor Ly49H^+^ engagement and thus lead to an efficient anti-viral NK cell response. This could explain why infection with either MCMV K181 or MCMV m157^G1F^ resulted in similar viral replication in B6 [13] and in Cmv1^r^ mice as shown here.

Analysis of m157 binding to NK cells revealed another degree of complexity in the parameters at play due to NK cell-borne MHC class I molecules able to interact in *cis* with Ly49C [21]. These *cis*-interactions impeded detection of Ly49C^+^ NK cells using the anti-Ly49C antibody 4LO3311 and prevented m157^G1F^ binding. This lack of binding was reversed by acid treatment. Identical binding patterns obtained with the 4LO3311 antibody and m157 are consistent with the findings that both bind the stalk domain of Ly49C [14], [20]. Ly49C binding to K^b^ in *trans* requires a back-folded configuration, while binding *in cis* requires an extended configuration [33]. Interestingly, our recent findings show that m157^G1F^ binds the Ly49C stalk region when the receptor adopts an extended configuration [14]. As the residues involved in Ly49C interactions with K^b^ *in cis* and m157 are distinct [14], one would predict that Ly49C may simultaneously bind both m157 and K^b^. However, our data indicate that these two interactions are mutually exclusive, as no m157 binding was detected when Ly49C was engaged by MHC class I *in cis*, and no inhibition of NK cell functions was achieved. Hence, Ly49C-dependent inhibition of NK cell response upon infection with MCMVm157^G1F^ was not as severe when the receptors could be masked *in cis* (infection of Ly49C Tg mice vs Ly49C Tg, β2m ko mice). It is possible that structural constraints in the Ly49C stalk domain that are incompatible with binding of m157 result from *cis*-binding to K^b^.

Inhibitory Ly49 receptors, such as Ly49C, contain intracellular immunoreceptor tyrosine-based inhibitory motifs (ITIM). When phosphorylated, ITIMs recruit phosphatases that subsequently suppress phosphorylation-based activation signaling [34]. *Trans*-binding to inhibitory Ly49 induces ITIM phosphorylation, however, it is likely that binding in *cis* does not generate such a tonic inhibitory signal. Rather, *cis*-interactions restrict the pool of Ly49 receptors that are available for functional interaction with ligands on target cells [33]. Our functional assays highlighted an important role for *cis* interaction in regulating anti-viral NK cell responses. Using B6 NK cells where cis-interactions largely prevent binding of m157 to Ly49C, we detected an un-inhibited Ly49H-dependent NK cell activation characterized by IFN-γ production and degranulation. Conversely, activation of the Ly49H^+^,C^+^ NK cell subset was almost completely abolished in B6 β2m ko NK cells where Ly49C/MHC *cis*-interactions are absent. These functional results correlated with the binding patterns of the m157-Fc and supported a role for MHC class I molecules in controlling Ly49C inhibitory functions in *cis*. To determine whether Ly49C could control the stability of cell conjugates formed through Ly49H engagement, we measured the formation of conjugates when Ly49H^+^ NK cells were exposed to m157-expressing targets and analyzed F-actin polymerization and polarization towards the targets; these events indicate NK cell activation and lead to formation of the cytolytic synapse that is required to mediate killing [35], [36]. Fewer conjugates were formed and cytoskeleton polymerization did not occur in B6 β2m ko NK cells expressing Ly49C. Thus, engagement of Ly49C in *trans* leads to inhibitory signals that destabilize cell conjugation and impairs cytoskeleton reorganization.

Our initial pathogenesis studies showed equivalent viral replication in B6 [13] and Cmv1^r^ mice infected with MCMV expressing m157^K181^ or m157^G1F^, despite the fact that m157^G1F^ binds both inhibitory and activating receptors on NK cells, while m157^K181^ binds only activating receptors. We produced a transgenic mouse in which all NK cells expressed Ly49C. Expression of the activating Ly49H receptor remained unchanged; however, these mice had an increased number of NK cells in the spleen. Despite the presence of more NK cells expressing Ly49H, infection of B6 Ly49C Tg mice resulted in higher viral loads compared to those seen in B6. Analysis of NK cells in infected B6 Ly49C Tg mice showed that only 30% displayed detectable Ly49C, consistent with the regulatory role played in *cis* by MHC class I molecules. To determine the effect of *cis*-masking on Ly49C-induced NK cell inhibition, we bred B6 Ly49C Tg with B6 β2m ko mice and generated B6 Ly49C Tg, β2m ko double mutant mice. These mice proved to be highly susceptible to MCMV infection and when infected with the usual dose of 5×10^4^ PFU of virus succumbed to infection within 3 days. This is consistent with a LD_50_ of 6.4×10^4^ PFU seen in BALB/c mice (that are devoid of Ly49H receptor) infected with the MCMV Smith strain [37] and suggests that Ly49C can completely obliterate Ly49H function in the absence of *cis*-masking. These results confirm the regulation exerted by *cis* interactions on NK cells functions *in vivo*.

Beside their inhibitory function in mature NK cells, inhibitory Ly49 receptors play a crucial role during NK cell education. NK cells acquire functional competence (are licensed) upon Ly49 interactions with MHC class I molecules [15]. Educated cells elicit stronger responses than NK cell subsets devoid of self-specific receptors [15], [16]. In B6 mice, licensing requires engagement of the inhibitory receptor Ly49C and signaling via the intracellular ITIM. Ly49C^+^ NK cells from B6 wt but not β2m ko mice produce IFN-γ in response to NK1.1 stimulation [15]. However, another study indicated that Ly49H^+^ NK cells robustly proliferate and produce IFN-γ in both B6 β2m ko and wt mice in response to MCMV infection [24]. We addressed NK cell responsiveness in situations where Ly49C plays a role (B6 origin) or not (B6 β2m ko origin) in NK cell education and confirmed that the NK cell response to MCMV does not require education through Ly49C. Furthermore, it was shown that unlicensed NK cells (Ly49C^−^) dominate the response to MCMV infection [17]. In B6 mice, Ly49H-dependent NK cell activation was shown to be stronger in the Ly49C/I^−^ subset, while Ly49C/I^+^ NK cells responded better to MHC class I deficient targets. Our experiments were conducted is similar settings to those described by Orr et al. albeit our target cells expressed m157^G1F^ and not m157^Smith^; we obtained similar IFN-γ production and degranulation in Ly49C^+^,H^+^ and Ly49C^−^,H^+^ NK cells from B6 mice. However, using B6 β2m ko NK cells, the Ly49C-expressing subset elicited a limited response, which was increased upon blocking of Ly49C using antibodies, even though it remained weaker than with the Ly49H^+^,C^−^ NK cells. These results suggest that the Ly49C^+^ subset had a lower intrinsic activity. The mechanisms responsible for the hyporesponsiveness of the Ly49C^+^ subset remain to be clarified.

Our data illustrated in figure 8A show a decreased killing of the untransfected RMA-S target cells when excess concentration of Ly49C blocking antibody was added to TC1 NK cells *in vitro*; this result was surprising because the blocking treatment should not interfere with binding to Ly49C of any ligand present on RMA-S in these settings. Instead, we propose that the decreased killing of RMA-S cells was due to a differential clusterization of the Ly49C receptor in untreated and anti-Ly49C treated NK cells. Indeed, it has been shown that restraints due to *cis* interactions limit the redistribution of Ly49A to the synapse, whereas unbound Ly49A dampens NK cell activation [38], [39]. Similarly, Ly49C receptors engaged in *cis* by MHC I molecules are likely to be contained in micro-clusters. Addition of excess concentration of blocking Ab may disrupt *cis* interactions, resulting in a re-localisation of the freed Ly49C to the immune synapse next to activating receptors where they can interfere with activating signaling pathways. Indeed, ITIM-bearing MHC I specific receptors such as Ly49C require activating receptors to be in the vicinity to perform their inhibitory function [40].

Interestingly, Ly49C engagement by m157^G1F^ had an inhibitory effect on a Ly49H-independent pathway, most likely due to “missing-self recognition” (Fig. 4 and 8). This activation pathway plays a role in NK cell mediated control of MCMV infection [30]. To counter this, MCMV encodes a number of proteins (m04, m06, m152) that interfere with MHC expression [41]–[43]. In mouse backgrounds where Ly49C is involved in NK cell education, such as B6 mice, Ly49C^+^ NK cells are expected to represent a major subset that responds to infected cells with reduced MHC class I expression. We found that MHC class I-deficient target cells expressing m157 (RMAS m157^G1F^) avoided killing by B6 NK cells *in vitro*. Similarly, *in vivo*, mice lacking Ly49H controlled MCMVΔm157 infection whereas the m157^G1F^-expressing virus escaped immune surveillance. We propose that infected cells displaying reduced MHC class I expression were ignored by NK cells as a result of m157^G1F^ binding to Ly49C. The response pattern we described here is not without analogies to that reported by Jonjic and colleagues [30] who showed that in BALB/c but not in BALB H-2^b^ mice, NK cells control an MCMV mutant lacking the m04 immunoevasin. NK cell activation was likely due to missing-self recognition, as NK cells in BALB/c β2m ko mice failed to keep viral replication in check. Babic proposed that m04 stabilizes MHC class I and improves binding to Ly49A to a lesser extent to Ly49C. In the absence of m04 inhibitory signals were reduced, which allowed a better elimination of the virus. This resembles our results obtained with MCMVm157^G1F^: inhibition was induced by m157 through Ly49C, whereas a Δm157 virus (like Δm04 virus) failed to induce inhibitory signals. We verified the m04 gene sequence in the MCMVm157^G1F^ recombinant virus and did not find any alterations (data not shown), which confirmed that the effects we noted were due to m157.

Our study provides a novel understanding of the parameters that direct how efficiently NK cells deal with viral infections. The variety of receptors encoded within the NKC, combined to a high level of sequence polymorphism between mouse strains, are critical determinants of whether NK cells can recognize and eliminate virus-infected cells. In addition, the host H-2 haplotype and MHC sequence polymorphism determine the possibility of interactions in *cis* that regulate NK cell receptor functions. Our *in vivo* results highlight why the stochastic expression of distinct NK cell receptors, which result in various NK cell subsets, is so important to host survival. In the ongoing race between the host and CMV, *cis*-binding of inhibitory NK cell receptors by MHC class I may have provided the host with a mechanism of countering viral immune evasion. Held and colleagues showed the role of *cis*-interactions between Ly49A and H-2 D^d^ in education and in regulating NK cell functions (reviewed in [33]). This mechanism could provide a strong evolutionary gain to mice that possess a suitable NK cell repertoire and specific sets of MHC class I molecules. We should also consider the possibility that an evolution-driven selection of individuals having developed Ly49 able to establish *cis*-interactions with MHC might have resulted from viruses producing immunoevasins able to inhibit NK cells through their self-specific receptors. A recent study demonstrated that the human inhibitory NK cell receptor LIR-1 interacts *in cis* with HLA-G; this affects the receptor accessibility for HCMV immunoevasin UL18 [44]. These results highlight the relevance of our work and its possible translation into human settings. Our findings provide novel insights into the mechanisms that dictate susceptibility to viral infections, such as HCMV, and thus may also indicate new immunotherapeutic approaches for regulating NK cell responses in settings of infection as well as possibly cancer and transplantation.

## Materials and Methods

### Ethics statement

All animal experiments were performed after approval by the Animal Experimentation and Ethics Committee of the University of Western Australia (AEC approval # RA/3/300/76 and RA/3/100/1079) and in accordance with the guidelines of the National Health and Medical Research Council of Australia (Australian code of the care and use of animals for scientific purposes 8^th^ edition, 2013; ISBN: 1864965975).

### Animals

Inbred C57BL/6 (B6) mice were obtained from the Animal Resources Centre (Perth, WA, Australia). BALB.B6-CT6 (H-2^d^, Ly49H^−^), BALB.B6-Cmv1^r^ (H-2^d^, Ly49H^+^) and B6.BALB-TC1 (H-2^b^, Ly49H^−^) congenic mice and B6 Ly49C Tg, B6 Ly49C Tg, β2m ko and B6 β2m ko recombinant mice were bred at the University of Western Australia Animal Care Services (Perth, WA, Australia). Female mice aged 7–12 weeks were used for all experiments, except for virus stock preparation where 3 week-old mice were used. All animal experiments were performed in pathogen-free conditions.

### Generation of Ly49C transgenic B6 mice

Splenic NK cells isolated from a B6 mouse were expanded in IL-2 for 12 d. Ly49C+ NK cells were sorted and mRNA was isolated using pureLink RNA minikit. A 907 bp long cDNA was amplified by PCR using the following primers Forward primer: CTC CAC CAG CAT CAC TCC G and Reverse primer: CAA GAA ACG AAT AAG GAT CAA CTC. A 850 bp product harboring Sal1 and BamH1 ends was generated by amplifying the ly49cB6 cDNA using the following primers Forward primer: TAT AT**G TCG AC**C TCC ACC AGC ATC ACT CCG and Reverse primer: TAT AT**G GAT CC**T TAA TCA GGG AAT TTA TCC. The ly49cB6 insert was inserted between the Sal1 and BamH1 restriction sites into the modified transgene cassette described in [45] and generously provided by Prof. W. Held (Ludwig Center for Cancer research, Lausanne, CH). The cassette was microinjected in fertilized C57BL/6 eggs (WEHI transgenic mouse facilities, Melbourne-Bundoora, VIC). Five founders were obtained and backcrossed to C57BL/6 mice. Appropriate offspring were identified by FACs analysis for Ly49C expression and checked by PCR; selected mice from a same dam were inter-crossed, four transgenic lines were generated among which one that displayed a homogenous Ly49C expression on over 95% NK cells was maintained.

### Cells and cell lines

BWZ.36, RMA and RMAS cells were grown in RPMI 1640 containing 5% FCS (Invitrogen), glutamine, sodium pyruvate, penicillin, gentamicin and 2-ME. COS-7 cells and Platinum-E cells were grown in DMEM (Invitrogen) containing 10% FCS, glutamine, sodium pyruvate, penicillin and gentamicin. Primary mouse embryonic fibroblasts (MEFs) were produced by trypsin dispersion of 15–17 day-old embryos from ARC/S mice as previously described [46]. MEFs and M210B4 cells were maintained in MEM 10% NCS (Invitrogen). Immediately prior to infection the culture medium was replaced with media supplemented with 2% FCS. For *in vitro* NK cell expansion, splenocytes were passed through a nylon wool column and then cultured in DMEM containing 10% FCS, glutamine, non essential amino acids sodium pyruvate, penicillin and gentamicin (all from Invitrogen) and 250 ng/ml recombinant human IL-2 (Cell Sciences, Canton, MA, USA).

### Production of m157-Fc soluble fusion proteins

The CP197 construct containing the Smith m157-Fc behind the CD150L sequence cloned in the CDM8 vector [9] was kindly provided by Prof. Lewis Lanier (University of California, San Francisco, CA USA). Soluble m157-Fc fusion proteins for the MCMV G1F isolate was constructed as described in [13].

### Virus stocks

The MCMV laboratory strains used were Smith (originally obtained from E.S. Mocarski, Stanford University) and K181-Perth (K181). Origin of wild-derived isolate G1F has been previously reported in [13]. Tissue culture virus stocks were produced by propagation in M210B4 cells and titers determined by standard plaque assay as previously described [47]. Virulent salivary gland viral (SGV) stocks were prepared by infecting 3-week-old female BALB/c mice i.p. with 1×10^3^ PFU of tissue-cultured passaged virus, and then preparing homogenates of salivary glands at 17 days post infection. Secondary SGV stocks were prepared by infecting 3-week-old female BALB/c mice with 1×10^3^ PFU of the primary SGV stock. Viral DNA was produced from infected MEFs as previously described [48].

### Construction of *m157* gene-swap MCMV viruses

In order to produce *m157* gene swap viruses we used a ‘BAC recombineering’ approach essentially as previously described [49]. The K181 BAC pARK25 DNA [50] kindly provided by A. Redwood (University of Western Australia). Generation of substitution mutants in which the K181 *m157* gene has been replaced with the G1F *m157* (MCMVm157^G1F^) sequences and deletion mutant in which the m157 gene has been deleted (MCMVΔm157) have been described in [13].

### Generation of BWZ transfectants

BWZ HD12 cells were generously provided by Prof. Wayne Yokoyama (Washington University, St. Louis, MO). BWZ HD12 reporter cells were transduced with the pMX-s-IRES-Ly49C^B6^ construct to generate BWZ HD12C cells as indicated in [13]. Clones expressing a range of levels of Ly49H^B6^ and Ly49C^B6^ were selected following limit dilution cloning, including clones which expressed essentially equal levels of these two receptors. For construction of Ly49H/C chimeric cells which express the Ly49H^B6^ transmembrane and cytoplasmic domains fused to the extracellular domains of Ly49C^B6^, Ly49H^B6^ was PCR amplified using forward primer (A): 5′-TATATCTCGAGATGAGTGAGCAGGAGGTCAC and reverse primer 5′-TCATTGATTTCTTGTTTGTGTTGA. Ly49C ^B6^ was amplified using forward primer 5′-CAAACAAGAAATCAATGAAACTCT and reverse primer (B): 5′-TATATGCGGCCGCTTAATCAGGGAATTTATCC. PCR products Ly49H^B6^ plus Ly49C^B6^ were sewed together by PCR using primers A and B above. The resultant PCR product was digested with XhoI and NotI and cloned into pMX-s-IG plasmid. The Plat-E packaging cell line was transfected with the plasmids and resulting retroviral supernatant used to transduce the BWZ-DAP12 reporter cells. Expression of Ly49C was confirmed by FACS.

### Antibodies

The antibodies used for analysis for flow cytometry were directed against the following proteins: CD3ε (145.2C.11), CD4 (RM4-5), CD8α (53.6.7), CD11b (M1/70), CD19 (1D3), CD44 (1M7), CD49b (DX5), CD69 (H1-2F3), CD107a (1D4B), H-2 IA/E (M5/114.15.2), IFN-γ (XMG1.2), mouse IgG1 (A851), Ly6C (AL21) and Fluorochrome-conjugated Streptavidin were obtained from BD BioScience (San Diego, CA). Anti-CD27 (LG7F9) and Siglec H (440c) were obtained from eBioScience (San Diego, CA). Anti-CD11c (N418) was obtained from BioLegend (San Diego, CA). Anti-human IgG (Fc part)-Biotin by Jackson ImmunoResearch Laboratories (West Grove, PA). Anti-Ly49H Ab 3D10 producing hybridoma was kindly provided by Prof. W.M. Yokoyama (Washington University, St. Louis, MO) and anti-Ly49C Ab 4LO3311 producing hybridoma was kindly provided by Prof. S. Lemieux (INRS, Laval, Canada). Purified anti-Ly49C and Ly49H antibodies were prepared in-house and conjugated to fluorochrome using antibody-labeling kit according to the manufacturer's recommendations (Molecular Probes).

### Staining for flow cytometry analysis

Staining with m157^G1F^-Fc fusion proteins was done as follows: cell lines or NK cells were incubated with m157^G1F^-Fc for 40 min, or shorter time when indicated, followed by anti human IgG (Fc part)-Biotin for 30 min and by a third step with Streptavidin-APC-Cy7 for 15 min. Non specific binding was provided in the absence of the initial incubation with the fusion protein. Live cells were gated based on exclusion of propidium iodide (PI, Sigma). Detection of Ly49C in B6 and Cmv1r NK cells was performed after release of the *cis*-interactions achieved by a mild acid treatment. Cells were washed twice in PBS and resuspended for 4 min at ambient temperature in Citrate buffer (0.133 M Citric acid, 0.066 M Na_2_HPO_4_, pH 3.3) at a density of 10.10^6^ cells/ml. The acid treatment was stopped by adding an excess of PBS 5% FCS; the cells were then stained for flow cytometry analysis. Acid treatment did not affect cell viability as determined by PI exclusion. For the detection of intracellular IFN-γ and of degranulation (LAMP1 expression), cells were cultured at 37°C in presence of monensin (Sigma-Aldrich, St Louis, MO). For LAMP-1 staining, anti-CD107a antibody (1D4B, BD Bioscience) was added into the culture medium as described in [51]. After the culture, cells were stained with fluorochrome conjugated antibodies directed to surface markers, the cells were then fixed with Cytofix/Cytoperm and permeabilized with Perm/Wash according to the manufacturer's recommendations (BD Bioscience). Permeabilized cells were incubated with anti-IFN-γ and anti-Ly49H antibodies for 40 min at room temperature. Intracellular detection of Ly49H was required to detect cells that had internalized this receptor upon exposure to m157-expressing target cells. Antibody-labelled cells were analyzed using a BD FACSCanto (BD Biosciences).

### Cell sorting

For *in vitro* assays, splenic cells were isolated and NK cells were expanded for 3–4 days in IL-2 containing medium; they were briefly treated in a mild acidic buffer, as above, and were stained with anti-NK1.1 (PK136), CD3ε (145.2C.11), Ly49H (3D10), Ly49C (4LO3311) fluorochrome-conjugated antibodies for cell sorting using a FACs Aria II cell sorter (BD Biosciences). Sorted NK cells were then placed back in IL-2 containing medium and expanded another 3 d to allow elimination of receptor-bound antibodies.

### Determination of virus growth *in vivo*

The recombinant viruses MCMVm157^G1F^, MCMVΔm157 and parental K181 MCMV virus were used to infect intra peritoneally BALB.B6-Cmv1^r^ mice at the dose of 1×10^4^ PFU and viral titers in the target organs (spleen, liver, lungs and salivary glands) were determined at designated times post inoculation (p.i.). The mice were sacrificed and organs were collected, homogenized in cold MEM 2% Neonatal Calf serum (NCS, Gibco) and centrifuged at 3000 rpm for 15 min at 4°C. The supernatants were stored at −80°C and viral titers were quantified by standard Plaque assay on M210B4 cells by standard plaque assay as described [47].

### Ly49H-independent NK cell response *in vivo*

B6.BALB TC1, BALB.B6-CT6 mice were infected intraperitoneally with 1×10^4^ PFU MCMVm157^G1F^ or MCMVΔm157; B6 β2m ko were infected with 1×10^4^ PFU MCMVΔm157. Infection was done in combination, or not, of an intraperitoneal injection of 2 µg α-galactosylceramide (αGC) (KRN7000, Kirin Brewer Co, Ltd, Japan). Mice were sacrificed 4 days p.i., the spleen, liver and lungs were collected and the viral titers within these organs was determined by standard plaque assay as indicated above.

### Conjugate assays

Sorted NK cells were labeled with 5 µM 5-(and -6)-(((4-chloromethyl) benzoyl) amino) tetramethyl-rhodamine orange (CMTMR, Molecular Probes, Carlsbad, CA) and target cells were labeled with 0.5 µM 5- (and 6-) carboxyfluorescein diacetate succinimidyl ester (CFSE, Molecular Probes) according to the manufacturer's recommendations. 10^5^ NK cells were mixed with 2×10^5^ targets in a final volume of 100 µl of DMEM 5% FCS and were centrifuged for 2 min at 50 g. After incubation at 37°C for 5 or 20 min, the conjugates were gently resuspended in 100 µl ice-cold 4% paraformaldehyde. Percentages of conjugated NK cells were then determined on a BD FACs Canto flow cytometer.

### Phalloidin staining

72 to 96 hours after sorting, Ly49H^+^,C^+^ and Ly49H^+^,C^−^ NK cells from B6 β2m ko mice were exposed to CMTMR labelled RMAS (top panels) or RMASm157^G1F^. 1×10^5^ sorted NK cells were mixed with 2×10^5^ CMTMR labeled targets in a final volume of 100 µl in DMEM 5% FCS and were centrifuged for 2 min at 50 g. After incubation at 37°C for 15 min, conjugates were gently resuspended and allowed to adhere on poly(L-lysine) coated microscopic slides (Lomb Scientific, Taron Point, NSW) for 3 min at 37°C and then were fixed with 4% paraformaldehyde for 10 min. After washing twice in PBS, slides were extracted with 0.1% Triton X-100 in PBS for 5 min at room temperature and were washed twice again in PBS. The slides were then stained with 1 unit of Phalloidin-FITC (Molecular Probes) diluted in PBS 1% BSA for 30 min at 37°C. After two washes in PBS, slides were mounted using Fluoromount (Sigma Aldrich) and analyzed on a fluorescence microscope. Polymerization of cytoskeleton actin as detected by phalloidin was quantified by using ImageJ software (National Institutes of Health; http://rsbweb.nih.gov/ij/); F-actin mean density was measured at the immune (IS) synapse and within the rest of a conjugated NK cell in order to determine the enrichment at the IS. A value close to 1 indicated that no polarization of the cytoskeleton toward the target cell was detected.

### Determination of NK cell cytotoxicity in *in vitro* assay

IL-2 activated NK cells were tested for cytotoxicity by standard ^51^Cr release assays [52]. Briefly, target cells were labeled with 100 µCi ^51^Cr (Perkin Elmer, Rowville, VIC Australia); radioactivity-labelled target cells were mixed with NK cells in triplicate at various E∶T ratios and incubated for 4 h at 37°C. Target cells incubated in Triton 1% provided the total radioactive content (maximal release) while targets cultured alone provided the spontaneous radioactive release values. Specific killing was determined by measuring the radioactivity released in cell-free supernatant according to the following formula: Specific killing = ((sample value – spontaneous release)/(maximal release – spontaneous release)×100). Radioactivity released in the culture medium upon target cell lysis was counted in cell-free culture supernatant using a Wallac Wizard γ-counter (Turku, Finland).

### Statistical analysis

For statistical analysis, the nonparametric Mann-Whitney test was performed using the statistical software package GraphPad Prism (La Jolla, CA, USA).

## Supporting Information

Figure S1 Analysis of m157^181^ and m157^G1F^ binding to Ly49H-expressing cells. (**A**) BWZ-Ly49H cells were incubated with increasing concentrations of m157^K181^-Fc or m157^G1F^-Fc for 40 min at 4°C and binding was measured by flow cytometry. The m157^G1F^-Fc concentration that achieved the highest mean fluorescence intensity (MFI) was used as reference (100%) and values obtained at lower concentrations were normalized according to the formula: % of binding = ((sample MFI – background MFI)/(maximal MFI – background MFI))×100). Results show MFI of triplicate values +/− SEM and are from one experiment representative of three. (**B**) BWZ-Ly49H cells were incubated with saturating concentrations of m157^K181^-Fc or m157^G1F^-Fc for periods ranging from 1 to 40 min at 4°C and the resulting binding was analyzed by flow cytometry. The MFI obtained at 40 min was used as reference (100%) and values obtained after shorter incubation times were normalized according to the formula: % of binding = ((timed MFI – background MFI)/(MFI at 40 min – background MFI))×100). Results show mean of triplicate values +/− SEM and are from one experiment representative of three. (**C**) BWZ-Ly49H cells were labeled with saturating concentrations of m157^K181^-Fc or m157^G1F^-Fc at 4°C; the cells were then washed and incubated at 37°C in the presence of excess concentrations of anti-m157 blocking antibody (6H121) to prevent re-binding of detached m157-Fc. A measure of the MFI prior to the addition of 6H121 provided maximal binding values (100%). Binding decay was measured at times from 5 to 90 min of incubation at 37°C. Binding decay values were calculated using the formula: % of binding = ((timed MFI – background MFI)/(MFI at 0 min – background MFI))×100). Results show mean of triplicate values +/− SEM are from one experiment representative of three.
(EPS)

Figure S2 Purified splenic NK cells from B6, Cmv1^r^ and B6 β2m ko mice were left untreated (left treated) or acid treated (right panel). Cells were stained with NK1.1, Ly49H and Ly49C antibodies to identify the four indicated subsets. Cells were incubated with anti-Fc biotin antibody, then with streptavidin-fluorochrome in the absence of a first step with m157-Fc to determine background fluorescence and evaluate the existence of non specific staining due to acid treatment. Horizontal bars represent the mean values of fluorescence measured from 3 individual mice (each represented as a circle). Data are form one experiment representative or three performed.
(EPS)

Figure S3 Populations of splenic leukocytes were analysed in naive B6 wt (n = 8) and B6 Ly49C Tg mice (n = 9) by flow cytometry. Differences between the groups were compared using Mann-Whitney U test: *: p<0.05, NS: not significant).
(EPS)

Figure S4 Expression of the Ly49C (**A,B**) and Ly49H (**C**) in splenic NK cells from naive B6 wt (n = 8) and B6 Ly49C Tg mice (n = 9) was analysed by flow cytometry. (**A**) For Ly49C detection, NK cells were previously acid stripped, or (**B**) were either acid stripped left untreated where indicated. Differences between the groups were compared using the Mann-Whitney U test: *: p<0.05, NS: not significant).
(EPS)

Figure S5 Populations of splenic leukocytes were analysed in naive B6 β2m ko (n = 5) and B6 Ly49C Tg β2m ko mice (n = 5) by flow cytometry. Differences between the groups were compared using the Mann-Whitney U test: *: p<0.05, NS: not significant).
(EPS)
